# Novel *ITGB6* Mutations Causing Amelogenesis Imperfecta

**DOI:** 10.3390/genes17040431

**Published:** 2026-04-08

**Authors:** Hyemin Yin, Soojin Jang, Hyuntae Kim, James P. Simmer, Jan C.-C. Hu, Jung-Wook Kim

**Affiliations:** 1 Department of Pediatric Dentistry, School of Dentistry & Dental Research Institute, Seoul National University, Seoul 03080, Republic of Korea; 2 Department of Biologic and Materials Sciences, School of Dentistry, University of Michigan, Ann Arbor, MI 48109, USA; 3 Department of Molecular Genetics, School of Dentistry & Dental Research Institute, Seoul National University, Seoul 03080, Republic of Korea

**Keywords:** whole exome sequencing, *ITGB6*, amelogenesis imperfecta, hereditary enamel defects, recessive mutation

## Abstract

Background/Objectives: Amelogenesis imperfecta (AI) is a heterogeneous group of rare hereditary conditions mainly affecting the quantity and/or quality of tooth enamel. Its phenotypic expression is diverse, as is the mutational spectrum of the AI-causing genes and mutations. Integrins are cell-surface receptors that mediate adhesion between cells and between cells and the extracellular matrix. Among these, mutations in integrin αvβ6 have been shown to cause AI; however, phenotypic variation exists between the knockout mouse model and human cases, as well as among different human AI families. Methods: We recruited AI families and performed mutational analysis using whole exome sequencing. Results: We identified compound heterozygous *ITGB6* mutations in two families. In Family 1, a paternally transmitted nonsense mutation (NM_000888.5: c.1060C>T, p.(Gln354*)) and a maternally transmitted missense mutation (NM_000888.5: c.2312A>G, p.(Asn771Ser)) were identified; in Family 2, a paternal missense mutation (NM_000888.5: c.1693T>C, p.(Cys565Arg)) and a maternal frameshift mutation (NM_000888.5: c.2091delC, p.(Asn698Metfs*13)) were identified, each causing AI in the respective proband. Both probands exhibited generalized hypoplastic and hypomineralized AI, but no other extraoral symptoms. Conclusions: This report will not only expand the known mutational spectrum of the *ITGB6* gene but also provide evidence for the genotype–phenotype correlations, thereby improving our understanding of the functional role of ITGB6 during amelogenesis.

## 1. Introduction

Tooth enamel is a mineralized tissue uniquely produced by epithelial cells, unlike other mineralized tissues (bone, dentin, and cementum), which are derived from mesenchymal precursors [1]. Enamel is composed of highly organized hydroxyapatite crystallites arranged into enamel rod and interrod structures. Amelogenesis, the process of enamel formation, is classically divided into four stages: presecretory, secretory, transition, and maturation [2]. Genetic alterations affecting tightly regulated, stage-specific developmental events can result in diverse enamel malformations with variable phenotypes. Depending on the developmental stage involved, these abnormalities may manifest as hypoplastic, hypomatured, hypocalcified, or mixed forms of enamel defect [3]. The group of inherited conditions characterized by abnormal enamel formation without comorbidities are defined as amelogenesis imperfecta (AI) [4].

More than 115 genes and disorders have been associated with hereditary enamel defects, reflecting the complex molecular regulation of enamel formation [5]. These genes encode proteins involved in diverse functions, including enamel matrix proteins (e.g., AMELX, ENAM, AMBN), enamel matrix proteases (MMP20, KLK4), mediators of cell–cell and cell–matrix adhesion (ITGB6, LAMA3, LAMB3, LAMC2, COL17A1, COL7A1, AMTN, ODAPH, FAM83H), ion transport and pH regulation during enamel maturation (WDR72, SLC24A4, GPR68, CNNM4, SLC13A5, CFTR), transcriptional regulation (DLX3, MSX2, SP6, CREBBP), mineralization regulation (FAM20A, FAM20C, VDR), intercellular communication (GJA1), paracellular transport (CLDN1, CLDN16, CLDN19), and ameloblast differentiation (SATB1) [6].

The integrin subunit beta 6 (*ITGB6*) gene (OMIM *147558) is located on the long arm of chromosome 2 (2q24.2) and spans slightly over 100 kb. Currently, there are seven reference mRNA transcript sequences that differ in the inclusion of 5′-most exons and in internal exon skipping; however, the last three exons are common to all. The representative mRNA sequence (NM_000888.5) consists of 15 exons, all of which are coding, and encodes a 788-amino-acid protein with a 21-amino-acid signal peptide. The integrin β6 subunit interacts with its heterodimeric partner, the αv subunit, to form the integrin αvβ6 cell-surface receptor, which plays an important role in epithelial cell adhesion and signaling [7].

The pivotal role of ITGB6 in tooth development was clearly demonstrated by *Itgb6*-null mice [8]. The null mice exhibited severe attrition and chalky, whitish anterior teeth instead of the normal yellow-pigmented, shiny appearance. Curiously, the expression of amelogenin, enamelin, and *KLK4* genes was significantly upregulated in the null mouse enamel organs. The enamel prism structure was defective, lacking a decussation pattern, and mineralization was severely impaired, consistent with enamel hypomineralization that resembles the human AI phenotype. Furthermore, these mice exhibited characteristic features of periodontal disease, suggesting that integrin αvβ6 contributes to the protection of periodontal tissues against inflammatory changes.

Interestingly, there are phenotypic differences between *Itgb6*-knockout mice and affected human subjects [9,10]. Human *ITGB6* mutations have been identified and their clinical enamel phenotype and associated symptoms have been characterized; however, the phenotypic spectrum and genotype–phenotype correlations remain incompletely defined [9,10,11,12,13]. In this study, we investigated two unrelated families with generalized rough hypoplastic/hypomineralized AI and identified novel *ITGB6* mutations contributing toward the understanding of ITGB6 regulation of amelogenesis.

## 2. Materials and Methods

### 2.1. Human Subjects

The Institutional Review Board of the University of Michigan and the Seoul National University Dental Hospital independently reviewed and approved the study protocol and consent forms (H03-00001835-M1 and CRI05003G). Informed consent was obtained from all participating family members or guardians. Study procedures—including explanation, pedigree construction, subject enrollment, clinical examinations, and saliva or peripheral blood sample collection—were conducted in accordance with the approved protocols and the Declaration of Helsinki.

### 2.2. Genomic DNA Characterization and Analysis

For the DNA extraction from the Family 1 family members, saliva samples (2 mL) were collected from each participant, and genomic DNA was extracted using the prepIT·L2P gDNA isolation kit (Norgen Biotek Corp, Thorold, ON, Canada). DNA quality was assessed by gel electrophoresis and quantified with a Qubit Fluorometer (ThermoFisher Scientific, Saint Louis, MO, USA). Genomic DNA from the proband underwent whole exome sequencing at the Johns Hopkins Center for Inherited Disease Research (CIDR) (Table 1). Variant annotation and mutational analyses followed established protocols [14]. Compound heterozygous *ITGB6* variants were identified during initial screening for candidate genes associated with hypoplastic/hypomineralized enamel defects and were subsequently confirmed by Sanger sequencing.

For the DNA extraction from the Family 2 family members, peripheral blood samples (2 mL) were collected from each participant, and genomic DNA was extracted using the NucleoSpin genomic DNA purification kit (Macherey-Nagel GmbH & Co., Düren, Germany). The purified DNA was measured for the quality and quantity by the NanoDrop^TM^ 1000 (ThermoFisher Scientific), a spectrophotometer, measured by the OD_260_/OD_280_ ratio. To further check the integrity of the isolated DNA, agarose gel electrophoresis was performed. Whole exome sequencing was performed at the BGI genomics (Shenzhen, China) (Table 1). Bioinformatic analysis, including sequencing reads trimming and alignment to the reference sequence and processing to get a list of short nucleotide variation and insertion/deletion, was performed as previously described [15].

### 2.3. Sanger Sequencing

PCR amplifications of *ITGB6* variants followed by Sanger sequencing for recruited family members were performed to determine segregation of the identified sequence variant with the disease phenotype (Table 2). PCR products were purified using PCR Purification Kit (QIAquick, Qiagen, Hilden, Germany and Elpis-Biotech, Daejeon, Republic of Korea) and sent for Sanger sequencing at the University of Michigan Advanced Genomics Core (Ann Arbor, MI, USA) or the DNA sequencing facility (Macrogen, Seoul, Republic of Korea).

## 3. Results

### 3.1. Family 1

**Figure 1:**
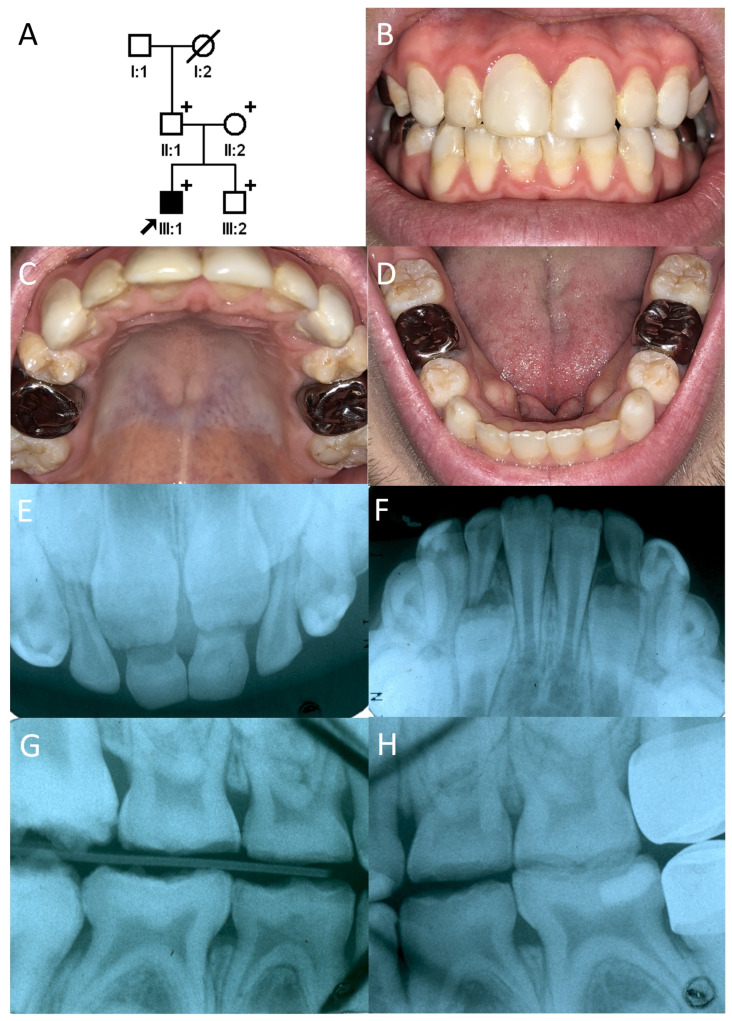
(**A**) Pedigree of Family 1. The black arrow denotes the proband. (**B**–**D**) Clinical photo of the proband. Anterior teeth and first molars are treated. Rough hypoplastic enamels can be seen in the premolars and second molars. (**E**–**H**) Intraoral periapical radiographs. Hypoplastic enamels are clearly seen in developing teeth. Hypoplastic enamel also exhibited less contrast with the underlying dentin indicating hypomineralization as well. The “+” signs denote study participants.

The proband was the first son of a non-consanguineous Caucasian family. He was evaluated at the Pediatric Dentistry Clinic, University of Michigan School of Dentistry, during the early mixed dentition stage, at approximately 6.5 years of age. Clinical findings-including enamel attrition on the occlusal surfaces and incisal edges, thermal sensitivity, as well as decreased enamel radiodensity-led to an initial impression of hypoplastic and hypomineralized AI (Figure 1). The family reported no significant medical history for the proband. The parents were initially offered participation in a genetic study, with detailed explanations of consent and assent forms, but declined. Fifteen years later, after the proband had become a dental student and learned about inherited dental defects, he persuaded his family to participate in the study. None of the other family members exhibited the phenotype observed in the proband (Figure S1).

**Figure 2:**
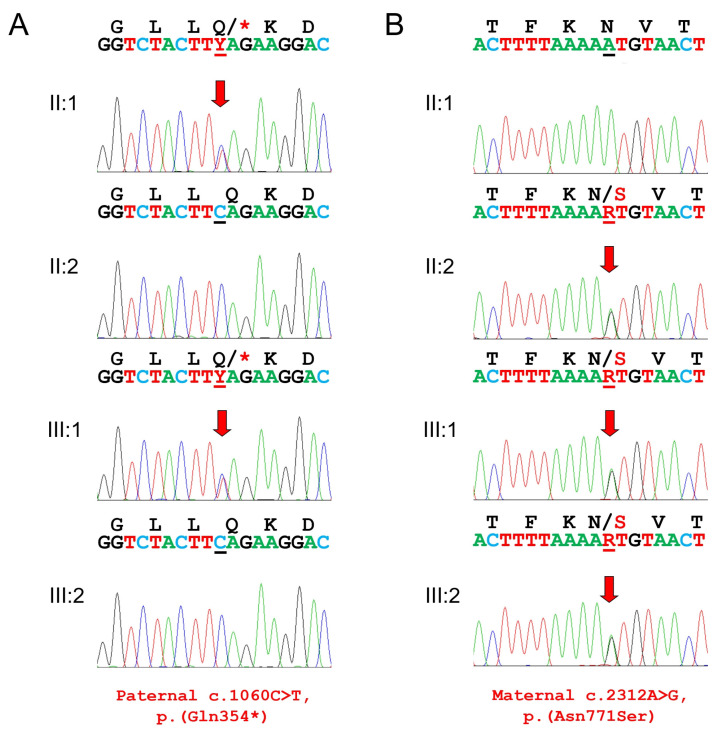
DNA sequencing chromatograms of the PCR amplification products. (**A**) Paternal nonsense mutation. (**B**) Maternal missense mutation. The amino acid sequence (**top**) and the nucleotide sequences (**bottom**) are shown above the chromatograms. Red arrows indicate the location of the mutation. Mutated nucleotides are underlined with black (wild-type allele) and red (mutant allele) lines. Asterisk denotes a stop codon.

**Figure 3:**
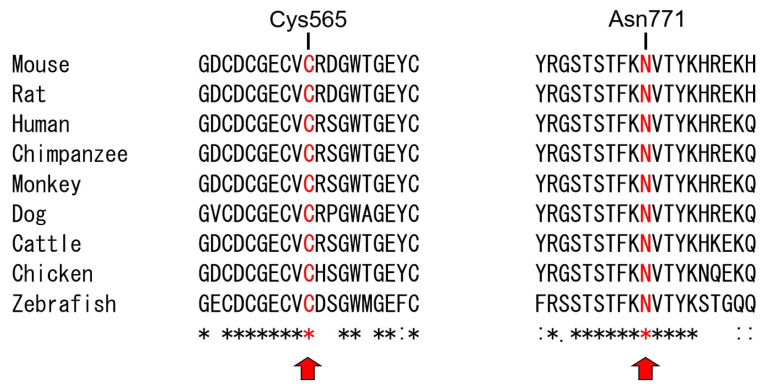
Sequences alignments of part of the *ITGB6* gene. Conservation of the Cys^565^ and Asn^771^ is indicated with red color in ITGB6 vertebrate orthologs. Conserved residues are denoted by asterisks (*), with colons (:) and periods (.) indicating high and low conservation, respectively.

The identified variants were a transition point mutation introducing an amber nonsense mutation in exon 8 of the *ITGB6* gene (NM_000888.5: c.1060C>T, p.(Gln354*)) and a transition point mutation in exon 15, the last exon, of the *ITGB6* gene substituting a highly conserved asparagine with serine at codon 771 (NM_000888.5: c.2312A>G, p.(Asn771Ser)) (Figure 2 and Figure 3). Segregation analysis showed that the nonsense mutation was paternal, whereas the missense mutation was maternal. The younger brother of the proband harbors only the missense mutation. The nonsense mutation was listed in dbSNP (https://www.ncbi.nlm.nih.gov/snp/ (accessed on 9 March 2026)) as rs770266214, with an allele frequency of 0.00003168 (51/1,609,740) in the Genome Aggregation Database (gnomAD) v4.1.0 (https://gnomad.broadinstitute.org/ (accessed on 9 March 2026)), but it was not listed in the ClinVar database (https://www.ncbi.nlm.nih.gov/clinvar/ (accessed on 9 March 2026)). The CADD (Combined Annotation Dependent Depletion, https://cadd.gs.washington.edu/score (accessed on 9 March 2026)) score was 40.0, indicating an extremely deleterious effect (scores ≥ 30). This nonsense mutation would likely undergo degradation via the well-known nonsense-mediated mRNA decay (NMD) pathway, which detects premature termination codon (PTC), rather than produce a truncated protein [16]. The missense mutation was not listed in dbSNP, and only two alleles were observed among 1,602,288 alleles in gnomAD v4.1.0 (allele frequency 0.000001248). In silico analyses unanimously predicted it to be pathogenic: the CADD score was 26, indicating a highly deleterious effect (scores ≥ 20); the PolyPhen-2 (http://genetics.bwh.harvard.edu/pph2/ (accessed on 9 March 2026)) score was 1.000, indicating a probably damaging effect (sensitivity: 0.00; specificity: 1.00); MutationTaster (http://www.mutationtaster.org/ (accessed on 9 March 2026)) predicted it to be disease-causing; and the MutPred (http://mutpred.mutdb.org/ (accessed on 9 March 2026)) score was 0.619, consistent with a likely pathogenic variant (scores > 0.5). MutPred further predicted an altered ordered interface with a probability of 0.35 (*p* = 0.0058).

### 3.2. Family 2

**Figure 4:**
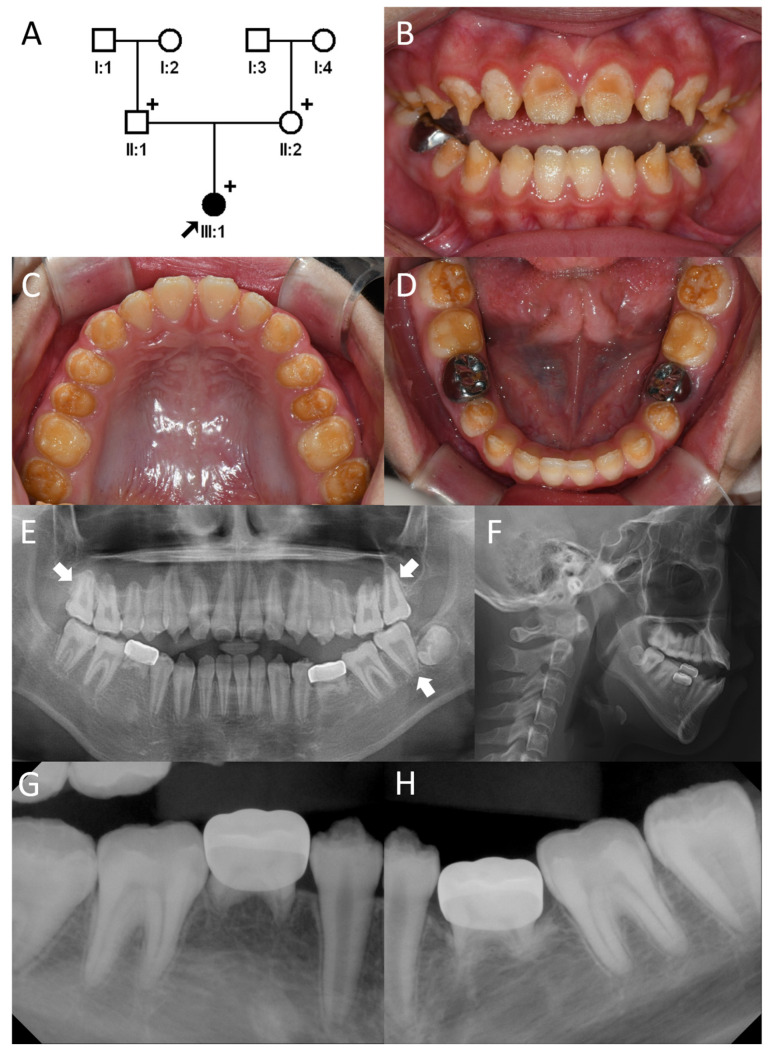
(**A**) Pedigree of Family 2. The black arrow denotes the proband. (**B**–**D**) Clinical photo of the proband. Generalized rough hypoplastic enamels with irregular brown discoloration can be seen in all teeth. (**E**) Panoramic radiograph exhibits taurodontism of second molars (white arrow). (**F**) Cephalogram shows anterior open bite. (**G**,**H**) Intraoral periapical radiographs exhibited hypoplastic and hypomineralized enamel. The “+” signs denote study participants.

The proband was a 12-year-old single daughter of a non-consanguineous Korean family (Figure 4). She visited the department of pediatric dentistry, Seoul National University Dental Hospital with the chief complaint of weak and unaesthetic dentition. She had rough hypoplastic teeth and irregular brown discoloration. She suffered from thermal sensitivity probably from the thin enamel coverage. She was born at 37 weeks and 6 days with a low birth weight (1.8 kg). She was discharged after 15 days in an incubator. Initially, her lungs were slightly underdeveloped and she had a tiny hole in her heart. The doctor said the hole was so small that it was acceptable to leave it as it was, and follow-up tests showed no abnormalities. She has never caught a cold or had pneumonia. She has never experienced shortness of breath or difficulty breathing, and she has tolerated exercise well. She was reported to have a mild allergy to buckwheat and some other foods. She presented with an anterior open bite, likely related to a prolonged bottle-feeding habit during early childhood. Radiographic examinations revealed that her enamel was not only hypoplastic but also reduced in mineralization.

**Figure 5:**
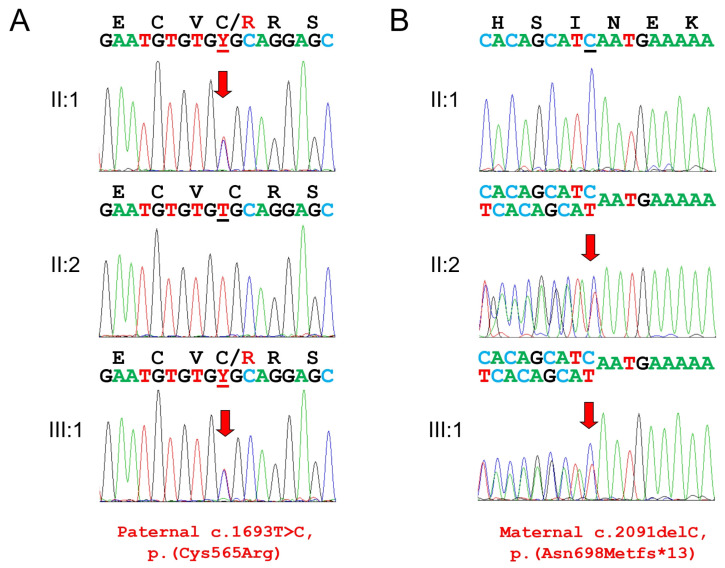
DNA sequencing chromatograms of the PCR amplification products. (**A**) Paternal missense mutation. (**B**) Maternal frameshift mutation. The amino acid sequence (**top**) and the nucleotide sequences (**bottom**) are shown above the chromatograms. Red arrows indicate the location of the mutation. Mutated nucleotides are underlined with black (wild-type allele) and red (mutant allele) lines.

The mutational analysis of the trio exome data revealed compound heterozygous *ITGB6* mutations in the proband (Figure 5). The identified variants consisted of a transition point mutation in exon 11, substituting a highly conserved cysteine with arginine at codon 565 (NM_000888.5: c.1693T>C, p.(Cys565Arg)) (Figure 3), and a single-nucleotide cytosine deletion in exon 13 (NM_000888.5: c.2091delC, p.(Asn698Metfs*13)). Segregation analysis showed that the missense mutation was paternal, whereas the frameshift mutation was maternal. The missense mutation was listed in the dbSNP database as rs780082370, with only two allele counts among 1,613,664 alleles (allele frequency 0.000001239) in gnomAD v4.1.0, but it was not reported in the ClinVar database. The CADD score was 28.4, the PolyPhen-2 score was 1.000 (indicating probably damaging, sensitivity: 0.00; specificity: 1.00), MutationTaster predicted it to be disease-causing, and the MutPred score was 0.962, indicating a very high-confidence pathogenic variant (score > 0.8). MutPred also predicted loss of the disulfide linkage at Cys565 with a probability of 0.47 (*p* = 0.00041). The maternal frameshift mutation was listed in the dbSNP database as rs768559532, with only one allele count among 1,612,598 alleles (allele frequency 0.0000006201) in gnomAD v4.1.0, but it was not listed in ClinVar. The CADD score was 34.0, indicating an extremely deleterious effect. The single-nucleotide cytosine deletion in exon 13 would introduce a PTC in exon 14. The PTC is located 137 nucleotides upstream from the exon junction complex between exon 14 and exon 15 (the last exon). Therefore, the mutant transcript would be degraded via NMD, rather than producing a truncated protein with 12 instead of the normal 91 amino acids at the C-terminus [17,18].

## 4. Discussion

ITGB6 forms a heterodimer with integrin αv (ITGAV) and binds to arginine-glycine-aspartic acid (RGD) motifs in extracellular matrix proteins, including fibronectin and the latency-associated peptide (LAP) of transforming growth factor-β1 (TGF-β1) [19,20]. Functional insights into ITGB6 have largely been derived from knockout mouse models, as its physiological role was initially unclear. *Itgb6*-null mice develop pulmonary inflammation and age-related emphysema associated with dysregulated macrophage *MMP12* expression [20,21]. Comparable systemic manifestations have not been consistently documented in human *ITGB6*-associated AI, possibly reflecting species-specific differences or limited clinical characterization [9,10,11,12,13].

Beyond its systemic epithelial functions, ITGB6 plays a crucial role in enamel development [8,22]. A central pathogenic mechanism of *ITGB6*-associated AI likely involves defective activation of TGF-β1. Binding of integrin αvβ6 to LAP induces TGF-β1 activation, which subsequently enhances *MMP20* expression through Runx2 signaling in ameloblast cell lines [23,24]. MMP20 is essential for the proteolytic processing of enamel matrix proteins, including amelogenin, during the secretory and early maturation stages of amelogenesis [25]. Disruption of this pathway impairs enamel matrix remodeling, leading to defective mineral deposition and structural enamel abnormalities [26]. Consistent with this, *Itgb6*-null mice exhibit chalky enamel, reduced mineralization, abnormal prism architecture, and severe attrition that recapitulate human hypomineralized AI [8]. However, because TGF-β1 activation and *MMP20* expression were unchanged in *Itgb6*-null mice, these findings suggest a distinct, as-yet-unproven mechanism may operate in humans versus mice, potentially resulting in different phenotypes.

Immunohistochemical analyses clearly demonstrate that ITGB6 is localized at the distal membrane of differentiating ameloblasts and pre-ameloblasts, with the strongest expression during the maturation stage, supporting its role in enamel mineralization and ameloblast modulation [9]. Therefore, *ITGB6* mutations could affect processes involved in the secretory and/or maturation stages, resulting in a hypoplastic and/or hypomineralized AI phenotype.

Structurally, integrins are composed of one α subunit and one β subunit, both of which are transmembrane glycoproteins comprising large extracellular domains and, in most cases, short cytoplasmic domains [22]. The extracellular head region of certain β subunits contains a βI domain, also referred to as the von Willebrand factor A (VWA) domain, which is essential for ligand binding and α-subunit interaction. Two domains, the integrin β epidermal growth factor-like (I-EGF) and the epidermal growth factor-like (EGF-like) domains, lie between the VWA and transmembrane regions [13,22]. Notably, all previously reported missense mutations are located within the VWA domain, underscoring its functional importance (Table 3). In this study, the proband of Family 1 had a paternal nonsense mutation (p.(Gln354*)) predicted to undergo NMD and a maternal missense mutation (p.(Asn771Ser)) in the short cytoplasmic domain. The proband of Family 2 carried a maternal frameshift mutation (p.(Asn698Metfs*13)) predicted to undergo NMD and a paternal missense mutation (p.(Cys565Arg)) located in the EGF-like domain. Collectively, these variants likely disrupt integrin αvβ6 stability or downstream signaling, supporting a shared loss-of-function mechanism.

Clinically, the anterior open bite observed in the proband of Family 2, as well as in previously reported *ITGB6*-related cases, broadens the phenotypic spectrum of *ITGB6*-associated AI [9,13]. Open bite occurs more frequently in individuals with AI than in the general population [27,28]. However, the precise pathophysiological mechanism underlying open bite in AI remains unclear. Prolonged oral habits, such as shielding hypoplastic teeth with the tongue or lips to minimize temperature sensitivity, may also contribute to the development of the open-bite phenotype.

Defining the precise clinical features of *ITGB6*-related AI remains challenging. While hypoplastic and/or hypomineralized enamel defects are consistently observed, the clinical presentation varies among affected individuals. A missense mutation identified in a Pakistani family resulted in pitted hypomineralized AI [11], whereas missense and nonsense variants reported in two Hispanic families were associated with generalized hypoplastic AI [9]. In a Turkish family, a missense mutation produced a phenotype characterized by pitted hypoplastic enamel with hypomineralization [10], while a nonsense mutation in a Thai family resulted in hypoplastic-hypomineralized AI accompanied by taurodontism [13]. Notably, another missense mutation reported in a Pakistani family presented only with a rough enamel surface, which is atypical of classic AI [12]. In the present study, both American and Korean families exhibited hypoplastic and hypomineralized AI, and taurodontism of the second molars was identified in Family 2. Given the limited number of reported cases and the genetic heterogeneity of human populations, such phenotypic diversity is not unexpected.

In summary, our findings reinforce the functional role of ITGB6 in enamel mineralization. The identification of novel *ITGB6* variants not only expands the mutational landscape of this gene but also highlights the phenotypic heterogeneity of *ITGB6*-related AI. Further functional studies and systematic clinical evaluations will be essential to refine genotype–phenotype correlations and to elucidate the broader biological implications of ITGB6 dysfunction.

## Figures and Tables

**Table 1:** Statistics for exome sequencing.

Sample	Total Reads	Mapping Rate (%)	Median Target Coverage	Coverage of Target Region (%)	Fraction of Target Covered with at Least	
					20×	10×
Family 1 II:1	112,522,367	98.8	93	99.5	96.9	98.8
II:2	102,578,191	98.4	85	99.4	95.6	98.5
III:1	113,144,599	99.0	91	99.5	96.4	98.7
III:2	103,473,916	96.5	81	99.5	95.9	98.5
Family 2 II:1	89,927,221	99.9	97	99.4	97.1	98.6
II:2	89,780,384	99.9	97	99.3	97.1	98.6
III:1	88,250,679	99.9	96	99.3	97.2	98.6

**Table 2:** Primers used for the Sanger sequencing.

Exon	Forward Primer	Reverse Primer	Size (bp)
8	5′-TAGACCATGGCAACCACAGA-3′	5′-TGGTGGATAGCCAACACTTG-3′	776
11	5′-GTCTTTTGATGACGGTGCTT-3′	5′-GGAGACCAAACCAGCAAATA-3′	441
13	5′-CCTATGCCTCTCCTATTCTCA-3′	5′-TTTGGAAATGTCTTTCCTGG-3′	360
15	5′-GGACTCAGTGCTGGGAAAAC-3′	5′-TGACTTTGCCGAGACAAAAA-3′	774

**Table 3:** Disease-causing mutations in *ITGB6* gene.

Location	cDNA	Protein	Mutation Effect/Domain	Mode of Inheritance	Classification	References
Exon 4	c.427G>A	p.(Ala143Thr)	Missense/ VWA domain	Paternal	Hypoplastic	Wang et al. (2014)
Exon 4	c.517G>C	p.(Gly173Arg)	Missense/ VWA domain	Homo	Hypoplastic	Seymen et al. (2015)
Exon 4	c.586C>A	p.(Pro196Thr)	Missense/ VWA domain	Homo	Pitted hypomineralized	Poulter et al. (2014)
Exon 5	c.625G>T	p.(Gly209*)	NMD	Maternal	Hypoplastic-hypomineralized	Sriwattanapong et al. (2023)
Exon 6	c.825T>A	p.(His275Gln)	Missense/ VWA domain	Maternal	Hypoplastic	Wang et al. (2014)
Exon 6	c.898G>A	p.(Glu300Lys)	Missense/ VWA domain	Homo	Rough/yellowish-brown stains	Ansar et al. (2016)
Exon 8	c.1060C>T	p.(Gln354*)	NMD	Paternal	Hypoplastic-hypomineralized	This report
Intron 10	c.1661-3C>G	Exon 11 skipping?	NMD	Paternal	Hypoplastic-hypomineralized	Sriwattanapong et al. (2023)
Exon 11	c.1693T>C	p.(Cys565Arg)	Missense/ EGF-like domain	Paternal	Hypoplastic-hypomineralized	This report
Exon 11	c.1846C>T	p.(Arg616*)	NMD	Homo	Hypoplastic	Wang et al. (2014)
Exon 13	c.2091delC	p.(Asn698Metfs*13)	NMD	Maternal	Hypoplastic-hypomineralized	This report
Exon 15	c.2312A>G	p.(Asn771Ser)	Missense/ Cytoplasmic domain	Maternal	Hypoplastic-hypomineralized	This report

## Data Availability

The data presented in this study are openly available in ClinVar (http://www.ncbi.nlm.nih.gov/clinvar (accessed on 17 March 2026)), Submission ID: SCV007518859, SCV007518860, SCV007518861, and SCV007518862.

## Supplementary Materials

The following supporting information can be downloaded at: https://www.mdpi.com/article/10.3390/genes17040431/s1, Figure S1: Intraoral photographs of the maxillary arch, centric occlusion, and mandibular arch in subjects II:1 (father), II:2 (mother), and III:2 (younger brother) of Family 1, each displaying permanent dentition.
