# Supplementary material for: Novel ITGB6 Mutations Causing Amelogenesis Imperfecta
Source: Genes (Basel). 2026 Apr 8;17(4):431. doi: 10.3390/genes17040431 (PMC13115778; doi:10.3390/genes17040431)
Supplement: Supplementary file 1 [file genes-17-00431-s001.zip › genes-4237683-supplementary.pdf]

II:1, II:2 and III:2 Intraoral Photos

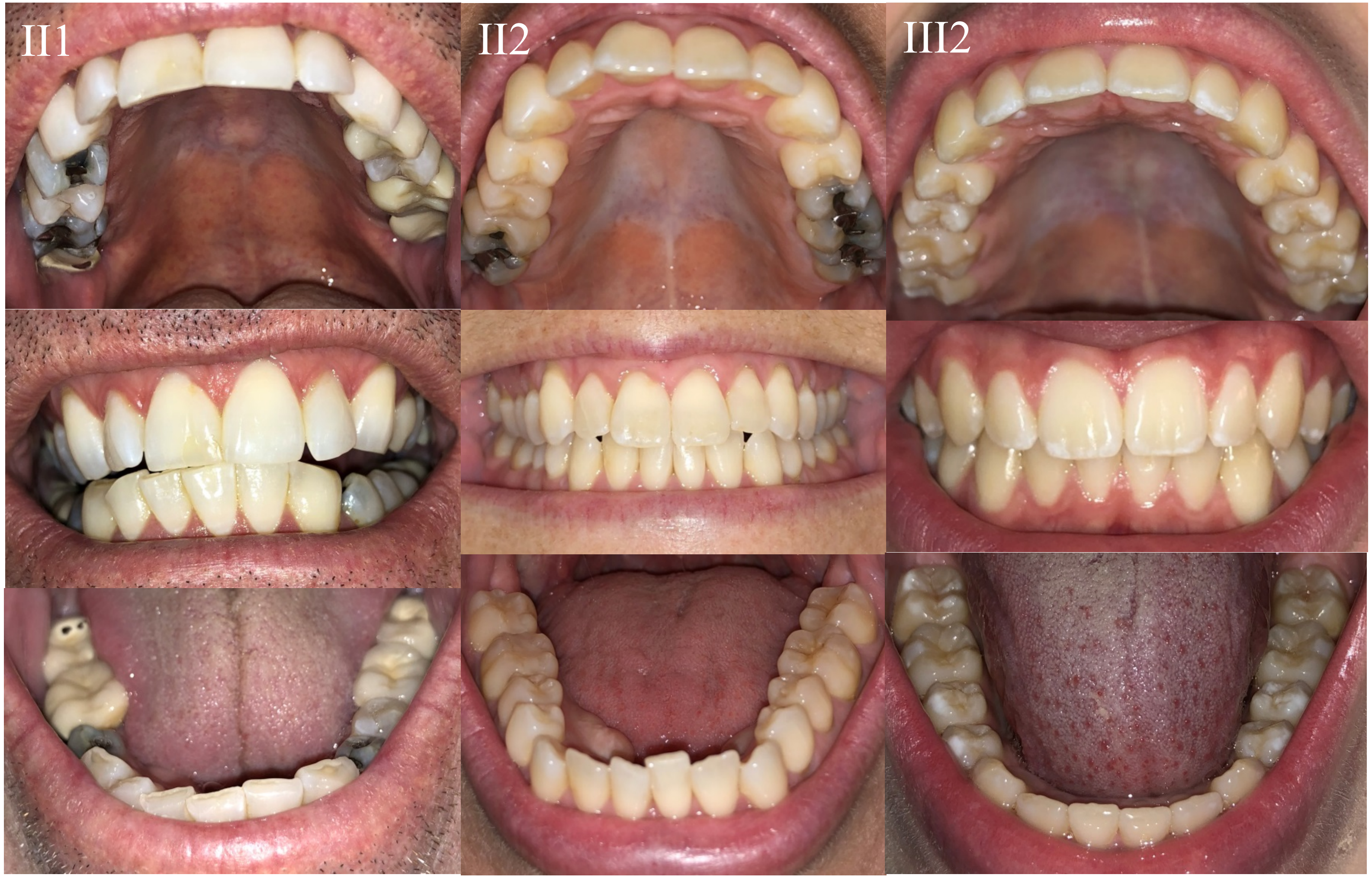

Figure S1. Intraoral photographs of the maxillary arch, centric occlusion, and mandibular arch in subjects II:1 (father), II:2 (mother), and III:2 (younger brother) of Family 1, each displaying permanent dentition. All individuals are unaffected.
